# A Newly Developed HPLC-UV/Vis Method Using Chemical Derivatization with 2-Naphthalenethiol for Quantitation of Sulforaphane in Rat Plasma

**DOI:** 10.3390/molecules26185473

**Published:** 2021-09-08

**Authors:** Kyong-Oh Shin, Kyungho Park

**Affiliations:** 1 LaSS Lipid Institute (LLI), LaSS Inc., Chuncheon 24252, Korea; 0194768809@hanmail.net; 2 Department of Food Science & Nutrition, and Convergence Program of Material Science for Medicine and Pharmaceutics, Hallym University, Chuncheon 24252, Korea; 3 The Korean Institute of Nutrition, Hallym University, Chuncheon 24252, Korea

**Keywords:** 2-naphthalenethiol, derivatization, sulforaphane, HPLC-UV/Vis, pharmacokinetics

## Abstract

Sulforaphane (SFN), a naturally occurring isothiocyanate, has received significant attention because of its ability to modulate multiple biological functions, including anti-carcinogenic properties. However, currently available analytical methods based on high-performance liquid chromatography (HPLC)-UV/Vis for the quantification of SFN have a number of limitations, e.g., low UV absorbance, sensitivity, or accuracy, due to the lack of a chromophore for spectrometric detection. Therefore, we here employed the analytical derivatization procedure using 2-naphthalenethiol (2-NT) to improve the detectability of SFN, followed by HPLC separation and quantification with UV/Vis detection. The optimal derivatization conditions were carried out with 0.3 M of 2-NT in acetonitrile with phosphate buffer (pH 7.4) by incubation at 37 °C for 60 min. Separation was performed in reverse phase mode using a Kinetex C18 column (150 mm × 4.6 mm, 5 μm) at a flow rate of 1 mL/min, with 0.1% formic acid as a mobile phase A, and acetonitrile/0.1% formic acid solution as a mobile phase B with a gradient elution, with a detection wavelength of 234 nm. The method was validated over a linear range of 10–2000 ng/mL with a correlation of determination (R^2^) > 0.999 using weighted linear regression analysis. The intra- and inter-assay accuracy (% of nominal value) and precision (% of relative standard deviation) were within ±10 and <15%, respectively. Moreover, the specificity, recovery, matrix effect, process efficiency, and short-term and long-term stabilities of this method were within acceptable limits. Finally, we applied this method for studying in vivo pharmacokinetics (PK) following oral administration of SFN at doses of 10 or 20 mg/kg. The C_max_ (μg/mL), T_max_ (hour), and AUC_0–12h_ (μg·h/mL) of each oral dose were 0.92, 1.99, and 4.88 and 1.67, 1.00, and 9.85, respectively. Overall, the proposed analytical method proved to be reliable and applicable for quantification of SFN in biological samples.

## 1. Introduction

Sulforaphane (SFN) is a naturally occurring sulfur-containing isothiocyanate enriched in natural products, such as broccoli, brussels sprouts, cauliflower, or cabbage [1,2]. SFN has received significant attention because of its ability to suppress cancer development or metastasis in multiple tissues through different mechanisms as follows: (i) modulation of carcinogen-associated enzymes, leading to suppression of the mutagenic activity within the target genes [3,4]; (ii) inhibition of cell growth and induction of apoptosis, thereby suppressing neoplastic development of the initiated and/or spontaneously transformed cells capable of producing cancer [5]; and (iii) inhibition of consequent angiogenesis for tumor progression and metastasis formation [6]. With this scientific evidence, investigators have been trying to determine the potential of SFN as an agent in cancer prevention and/or therapy [7].

Accordingly, downstream studies for assessing the pharmacokinetics (PK) and pharmacodynamics (PD) profiles of SFN are required for both preclinical and clinical stages of the drug discovery process. Although prior studies have reported methods for quantifying SFN utilizing high-performance liquid chromatography (HPLC)-UV/Vis [8,9,10] or LC-mass spectrometry (MS) [11,12], the current analytical methods are still not in common use in the quantification of SFN due to a number of reasons: (1) the HPLC-UV/Vis detector method has poor sensitivity because SFN has no UV chromophore; (2) although the LC-MS-based method has better capability in accurate identification and sensitive detection compared with HPLC methods, this methodology requires expensive instruments and highly trained personnel, suggesting that the LC-MS-based analytical method cannot be used for small-scale laboratory research. Therefore, alternative methods that can provide high sensitivity and are cost effective are needed to fulfill the requirements of quantification or qualification in SFN-mediated studies, including PK/PD. Recent studies revealed that chemical derivatization with appropriate reagents before an HPLC-equipped UV/Vis detector is generally used to determine certain analytes lacking chromophores, including SFN. Of different derivatizing reagents, such as Cys-ME [8], mercaptoethanol [11], benzenethiol [13], or 2-naphthalenethiol (2-NT) [14], 2-naphthalenethiol (2-NT) exhibits high molar absorptivity and longer and/or specific wavelengths (≥230 nm; 234, 280, and 320 nm), and sensitive detection with reduced interference from the sample matrix can be achieved in comparison to others [14]. Despite such benefits, no studies have employed 2-NT as a derivatizing reagent for SFN quantification measured by the HPLC-UV/Vis method.

In the present study, therefore, we utilized the chemical derivatization reaction using 2-naphthalenethiol (2-NT) to improve the detectability of SFN, followed by the development of specific HPLC-UV/Vis analytical conditions and justified each validation characteristic within acceptable limits. Moreover, we successfully applied the proposed method to study rat pharmacokinetics (PK) following oral administration of SFN.

## 2. Results and Discussion

### 2.1. Method Development

**Figure 1:**
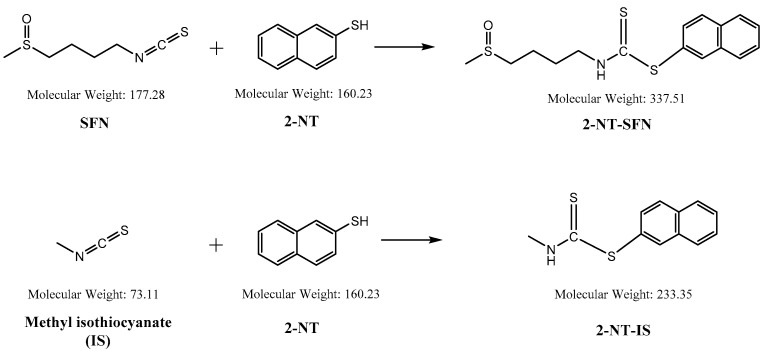
Chemical structures of sulforaphane (SFN) and methyl isothiocynate employed as an internal standard (IS), and their derivatives reacted with 2-naphthalenethiol (2-NT).

**Figure 2:**
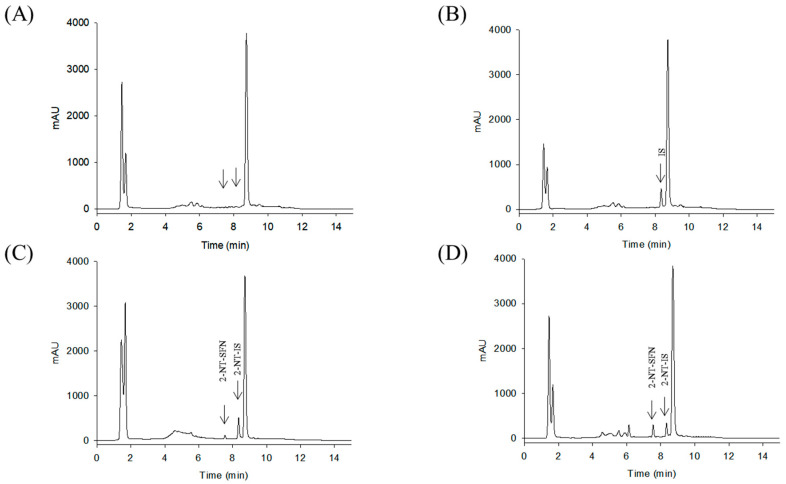
Representative HPLC chromatograms of double blank rat plasma (**A**), blank rat plasma spiked with the IS (**B**), blank rat plasma spiked with 0.01 μg/mL of SFN (LLOQ) or the IS (**C**), a plasma sample obtained 1 h after a single oral administration of 10 mg/kg of SFN (**D**). SFN, sulforaphane; 2-NT, 2-naphthalenethiol.

**Figure 3:**
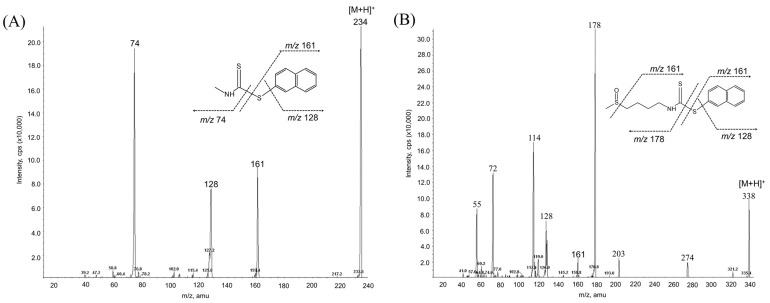
MS/MS spectra of 2-NT-SFN (**A**) and 2-NT-IS (**B**) obtained by ESI in positive mode using a collision energy of 30 eV. IS, internal standard; SFN, sulforaphane; 2-NT, 2-naphthalenethiol.

While the reaction of isothiocyanates (ITCs) derived from natural sources, such as SFN, with thiol leads to the formation of a stable dithiocarbamate ester, several prior studies have used certain organic reagents, e.g., mercaptoethanol [11] or N-tBOC-Cys-ME [8] or benzenethiol [13], for the detection and quantification of ITCs. However, as previously developed methods for SFN quantification still have a number of weaknesses, e.g., low sensitivity, accuracy, or cost effectiveness, we here developed a new method for quantitation of SFN using HPLC coupled with a UV/Vis detector after analytical derivatization with 2-naphthalenethiol (2-NT). The derivatization process of SFN or its internal standard (IS) isothiocynate using 2-NT is shown in Figure 1. The derivatization condition was optimized by changing various parameters, i.e., the concentration of the derivatization agent 2-NT (0.05–0.5 M), range of pH (4.0–10.0), incubation temperature (25 to 60 °C), and period of incubation (10–180 min). As such, the optimal derivatization conditions were obtained with 0.3 M of 2-NT in acetonitrile with phosphate buffer (pH 7.4) by incubation for 60 min at 37 °C. A typical chromatogram of SFN and IS after chemical derivatization with 2-NT is shown in Figure 2. We confirmed separated symmetric peaks for 2-NT-SFN and 2-NT-IS at a run time of 7.5 and 8.4 min, respectively. Moreover, the selectivity and specificity of the proposed analytical method were evaluated by the absence of any endogenous interference at the retention times of peaks of interest as evaluated by the chromatograms of the following samples: the blank rat plasma (Figure 2A), blank rat plasma spiked with the internal standard (Figure 2B), blank rat plasma spiked with 0.01 μg/mL of SFN (LLOQ) and the internal standard (Figure 2C), and the plasma collected from a rat 1 h following a single oral administration of 10 mg/kg SFN or IS (Figure 2D). Next, we further confirmed the derivatives of both SFN and IS using ESI-MS/MS in positive mode with 30 eV of collision energy. While fragment ions of 2-NT-SFN were generated at *m*/*z* 338, 274, 178, 161, 128, 114, and 72, we identified that ions at *m*/*z* 274, 178, 114, 72, and at *m*/*z* 161, 128 corresponded to SFN and 2-NT moieties, respectively (Figure 3A). In addition, fragment ions of 2-NT-IS were at *m*/*z* 338, 161, 128, 74.0, while ions at 161, 128 *m*/*z* stand for 2-NT (Figure 3B). To quantify the derivatized SFN or IS, the chromatographic condition was examined by altering a number of parameters, such as buffer, organic solvent, and absorption spectra, for the detection. The optimized separation was carried out with 0.1% formic acid (solvent A) and acetonitrile with 0.1% formic acid (solvent B). In addition, the highest signals of the derivatives were specified at the wavelengths of 234 nm under UV/Vis measurements.

### 2.2. Method Validation

#### 2.2.1. Linearity and Calibration Curve

The equations of coefficients of determination (R^2^) and linear regression are described in Table 1. R^2^ of three replicates was greater than 0.999, and the accuracy of all three calibration points was within ±10% of the nominal concentration, indicating excellent linearity with a detection range of 10–2000 ng/mL. The regression equation was y = 2.2502x + 0.0499. The limits of detection (LOD) and limits of quantification (LOQ) were 0.0028 and 0.0091, respectively (Table 1), suggesting that the detection range and sensitivity of calibration curves of our method were sufficient to quantify SFN levels in rat plasma after the single dietary doses of 10 or 20 mg/kg SFN.

#### 2.2.2. Accuracy and Precision

The results of the intra- and inter-assay accuracy and precision are described in Table 2. The accuracy and precision of all the QCs at different levels were within ±10%, and <15%, respectively. In addition, the mean accuracy and precision values obtained were 92.15 and 8.14%, respectively. Our accuracy and precision results satisfied the USFDA guidelines for bioanalytical method validation, suggesting that rat plasma samples with a concentration >10 ng/mL of SFN can be quantified with good enough accuracy and precision.

#### 2.2.3. Recovery and Matrix Effect

Table 3 summarizes the results of the matrix effect and extraction recoveries of SFN and IS from rat plasma samples. The mean extraction recoveries and efficiency at three different levels of SFN and IS were ±15, ±18% or ±20, ±22%, respectively, indicating a good recovery in different rat plasma samples. The mean matrix effects at three different levels of SFN (0.03, 0.8, and 2.0 µg/mL) were −2.87, −2.99, and −2.37%, while the IS at 0.5 µg/mL was −2.58. These results indicated the presence of some matrix effect in terms of the 2-NT-SFN/2-NT-IS response ratio. Nevertheless, because the LLOQ signal intensity was sufficient for the SFN quantification, this method can be applied for downstream analysis, such as the PK study.

#### 2.2.4. Stability

The results of the short-term and long-term stabilities of SFN in rat plasma are summarized in Table 4. SFN stock solutions were stable for at least 3 weeks when prepared in an acetonitrile solution at −20 °C (data not shown). Moreover, nominal% of QCs at three concentrations (0.03, 0.8, or 2 μg/mL) of SFN after 30 days at −20 and −80 °C were within ±19% or ±11%, respectively. These results indicate that SFN is stable for at least 30 days in rat plasma under the described storage conditions (Table 4).

### 2.3. In Vivo Pharmacokinetics Study

**Figure 4:**
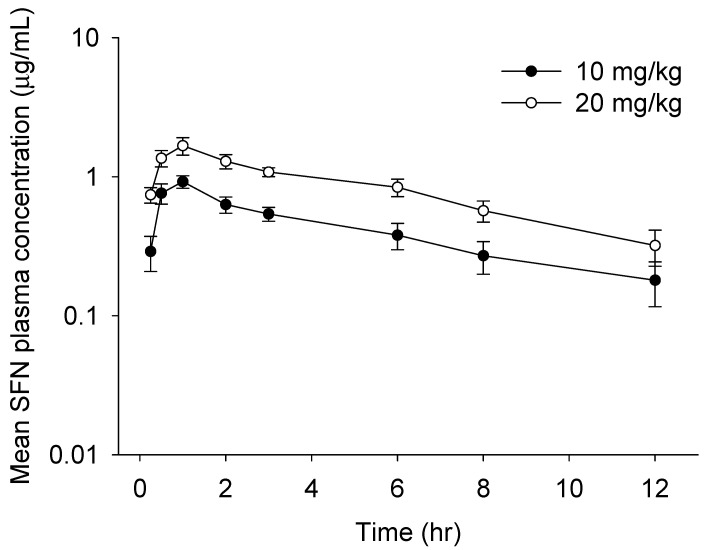
Mean plasma concentration–time plot of sulforaphane (SFN) after a single oral administration of SFN at 10 or 20 mg/kg to rats. All values are mean ± SD (*n* = 4 rats).

We finally performed in vivo pharmacokinetics (PK) study of oral SFN using a newly developed method. The mean plasma concentration–time profiles and PK parameters calculated by the non-compartment model of SFN in rat plasma are described in Figure 4 and Table 5, respectively. The concentration of SFN was readily measurable in plasma samples collected up to 12 h post-dose. Consistent with prior findings [9,15], the maximum plasma concentrations of oral SFN (10 mg/kg: 0.92 μg/mL; 20 mg/kg: 1.67 μg/mL) were reached in approximately 1 h at both doses, suggesting that SFN is rapidly absorbed from the gastro-intestinal tract. A rapid absorption rate could be caused by the chemical properties of SFN, i.e., a low molecular weight and higher lipophilicity. Afterwards, SFN at both doses was eliminated with a half-life of approximately 5–6 h, which is consistent with previous findings that SFN is rapidly eliminated after oral absorption [9,15,16]. Finally, our studies revealed that the values of AUC_0–12h_, AUC_0–∞h_, AUMC, and C_max_ in orally administrated rats increased in a dose-dependent manner, suggesting that a newly established method would be a time-efficient and accurate method for measurement of SFN in rat plasma applicable for in vivo PK study.

## 3. Materials and Methods

### 3.1. Chemicals and Standards

Sulforaphane (SFN) (>90% purity), methyl isothyocyanate (>97% purity) as an internal standard (IS), formic acid (analytical reagent grade), and 2-naphthalenethiol (99% purity) were purchased from Sigma-Aldrich (St. Louis, MO, USA). Organic solvents, such as methanol, ethyl alcohol, acetonitrile, and chloroform, for both SFN extraction and HPLC analysis were supplied by Merck (Darmstadt, Germany). Purified deionized water was obtained from an in-house purification system (18 MΩ, Millipore, Bedford, MA, USA). Unless otherwise stated, all other chemicals were obtained from Sigma (St. Louis, MO, USA).

### 3.2. Equipment

The Agilent 1260 series HPLC system (Agilent Technologies, Santa Clara, CA, USA) was equipped with an auto-sampler, degasser, quaternary pump, and UV/Vis detector. Analyte separation was performed in reverse phase mode with a Kinetex C18 column (150 mm × 4.6 mm, 5.0 μm). Data manipulation was performed using the Chemstation B.04.03 software.

### 3.3. Preparation of the Stock Solution and Working Solutions

SFN was dissolved in acetonitrile to prepare stock solutions at a concentration of 1000 μg/mL. Working solutions at concentrations of 0.01, 0.025, 0.05, 0.1, 0.25, 0.5, 1, and 2 µg/mL were obtained by serial dilution from stock solution by adding an appropriate volume of ethyl alcohol (EtOH). Methyl isothiocyanate, which was employed for the internal standard (IS), was diluted with acetonitrile to obtain the working IS solution at a concentration of 5 μg/mL. All stock solutions were stored at −20 °C and protected from light.

### 3.4. Calibration Standards and Quality Control (QC) Samples

Calibration standard working solutions were diluted 10-fold with rat blank plasma (1:9, *v*/*v*) to obtain 6 calibration standards at 0.01, 0.025, 0.05, 0.1, 0.25, 0.5, 1, and 2 µg/mL. Quality control (QC) working solutions were also diluted 10-fold with rat blank plasma to obtain QCs at 4 different concentration levels, i.e., low, mid, and high levels of 0.01, 0.03, 0.8, and 2.0 µg/mL, respectively.

### 3.5. SFN Extraction from Rat Plasma and Chemical Derivatization with 2-NT

In total, 100 μL of blank rat plasma, rat plasma spiked with SFN, or plasma collected from rats orally administrated SFN at doses of 10 or 20 mg/kg for the pharmacokinetic (PK) study were mixed with 10 μL of the IS working solution, followed by incubation at room temperature for 3 min. The reacted solution was then mixed with the solution of saturated sodium chloride and chloroform (1:4, *v*/*v*), and centrifuged at 10,000× *g* for 5 min. The organic phase was separated from the aqueous layer, and dried using a speed vacuum system (Vision, Seoul, Korea). The residue dissolved in ethanol was further derivatized with 0.3 M 2-naphthalenethiol (NT) by incubation at 37 °C for 60 min, and finally the reacted residue was fltered using a 0.45 μm PTFE filter (Millipore, Billerica, MA, USA) prior to HPLC analysis.

### 3.6. Derivatives Confirmation by ESI Mass Spectrometry

An API 3200 triple quadruple Mass Spectrometer (Applied Biosystems, Foster City, CA, USA) equipped with an electrospray ionization (ESI) interface in positive ionization mode was employed for the confirmation of SFN derivatives. The mass range was set between 50 and 500 *m*/*z* and derivatives were infused directly into the mass spectrometry. The optimized instrument conditions were as follows: source temperature, 400 °C; curtain gas pressure, 20 psi; nebulizing gas (GS1) pressure, 50 psi; and heating gas (GS2) pressure, 40 psi. The first quadrupole (Q1) was set to unit resolution and Q3 to low resolution. Analyst software (ver. 1.4.2; Applied Biosystems, Foster City, CA, USA) was used for instrument control and data collection.

### 3.7. Chromatographic Conditions to Quantify SFN in Rat Plasma

The LC chromatographic separation was conducted with a Kinetex C18 column (150 mm × 4.6 mm, 5.0 μm). The mobile phase was delivered at a flow rate of 1.0 mL/min through gradient elution and consisted of pure water with 0.1% formic acid (aqueous mobile phase A) and acetonitrile with 0.1% formic acid (organic mobile phase B). The total analytical run time for each injection was 15 min, including 5 min of re-equilibration.

The initial gradient elution started with 10% mobile phase B, which was maintained for 1 min, followed by gradual elevation of mobile phase B to 70% over 5 min. These experimental conditions were held for 3 min, returned to initial conditions, and the column was re-equilibrated for 5 min. Optimization of chromatographic conditions involved the subsequent evaluation of the following parameters: buffer, mobile phase, organic solvent, gradient elution, flow rate, auto-sampler temperature, column temperature, and injection volume. Finally, the conditions showing the best retention and separation were then selected. Both SFN and IS derivatives’ absorbance was detected at 234 nm. Research manuscripts reporting large datasets that are deposited in a publicly available database should specify where the data have been deposited and provide the relevant accession numbers. If the accession numbers have not yet been obtained at the time of submission, please state that they will be provided during review. They must be provided prior to publication.

Intervention studies involving animals or humans, and other studies that require ethical approval, must list the authority that provided approval and the corresponding ethical approval code.

### 3.8. Method Validation

Full validation of the current method in rat plasma was performed in accordance with the guidelines of the US Food and Drug Administration (FDA) on chromatographic bioanalytical method validation. The validation included the following parameters: linearity, stability, selectivity, sensitivity, and intra-/inter-day accuracy and precision. The linearity of this method was evaluated using calibration standards prepared at six concentrations over a range of 0.01–2.0 μg/mL. Repeatability or intra-assay accuracy were determined by analyzing five individually prepared replicates at each concentration within the same run and five injections of one replicate within another run to evaluate injection repeatability. Inter-assay accuracy and precision were obtained by analyzing five individually prepared replicates at each concentration within five different days. The stability of SFN in rat plasma was determined using five individually prepared replicates of QCs at three concentration levels. The following stability conditions were evaluated: short-term stability (24 h at room temperature, 4 °C and at −80 °C), post-preparation with or without exposure to derivative reagent (24 h or 7 days at 4 °C), freeze thaw (three cycles, −20 °C/room temperature, and 24 h between cycles), and long-term stability (30 days at −20 and −80 °C).

### 3.9. In Vivo Pharmacokinetic Study

All animal procedures were approved by the Institutional Animal Care and Use Committee (IACUC) of Hallym University (Permit number: Hallym2020-48) and performed in accordance with their guidelines as well as ARRIVE guidelines (Animal Research: Reporting of In Vivo Experiments) (https://www.nc3rs.org.uk/arriveguidelines; accessed on 15 February 2021). Eight-week-old Sprague–Dawley (SD) female rats, weighing about 230–250 g, were purchased from DBL Ltd. (Eumseong, Korea) and used for the pharmacokinetic study. All experimental rats were housed in individual cages at the Hallym University Laboratory Animal Resources Center under specific pathogen-free (SPF) conditions with a controlled consistent temperature (23 ± 2 °C) and lighting environment (12 h/12 h light/dark cycle). At the end of the study, the experimental mice were sacrificed by CO_2_ inhalation. A gradual fill rate of 20% chamber volume per minute of displacement was used for CO_2_ euthanasia. All efforts were made to minimize the number and suffering of any animals used in these experiments. Animals were randomly divided into two groups after an acclimation period of 1 week. SFN dissolved in water was administered as a single oral dose of either 10 mg/kg or 20 mg/kg. Blood samples (300 μL) were collected in lithium-heparinized tubes from the tail vein before dosing and subsequently at 0.25, 0.5, 1, 2, 3, 6, 8, and 12 h after administration. Blood samples were then centrifuged at 10,000× *g* for 3 min at 4 °C to separate plasma. The SFN pharmacokinetic parameters processed by non-compartmental analysis of plasma concentration versus time data using the computer program Winnonlin Ver. 5.1 (Pharsight Corporation, Mountain View, CA, USA) were as follows: the area under the plasma concentration–time curve to the last measurable plasma concentration (AUC_0–t_); the area under the plasma concentration–time curve to time infinity (AUC_0–∞_); the maximum plasma concentration (C_max_); the time to reach the maximum concentrations (T_max_); the elimination half-life (t_1/2_); the mean residence time (MRT); and total plasma clearance (CL). Both C_max_ and T_max_ were obtained directly from the generated curve.

## 4. Conclusions

A new optimized HPLC-UV/Vis method for the quantification of SFN in rat plasma was developed and validated in accordance with USFDA guidelines. To the best of our knowledge, this is the first study to utilize 2-NT derivatization for a HPLC-UV/Vis-based quantification of SFN. While prior studies established the HPLC-UV/Vis methods without chemical derivatization [9,17,18,19], which exhibited that UV absorbance at a short wavelength (202–210 nm) causes a relatively high background and low sensitivity, the use of 2-NT derivatization significantly increases the UV absorption at a wavelength of 234 nm, helping to improve the detectability of SFN. Moreover, the advantage of this method lies in its low limit of detection (LOD) of 0.0078 µg/mL compared to a previous HPLC assay with an LOD of 0.01 µg/mL [9]. The assay was fully validated, with good selectivity and linearity over a large range of 0.01–2.0 µg/mL. SFN was stable in the solvent employed in this study and plasma for at least 6 months and 1 month, respectively. Moreover, we successfully applied this method to the in vivo pharmacokinetic study of SFN with single oral dietary doses. A limitation of this study is the absence of clinical application on plasma samples of patients receiving SFN administration. Thus, future studies are needed for assessment of the assay using patients’ plasma samples. Overall, the method we developed in the present study could be useful to perform not only clinical PK/PD studies but also to investigate SFN side effects, which will be instructive in the creation of a dosage regimen and optimization of SFN safety and efficiency.

## Figures and Tables

**Table 1:** Summary of the calibration curve, linear range, LOD and LOQ for the quantification for SFN in rat plasma by the HPLC method.

Calibration Curve	R^2^	Linear Range (µg/mL)	LOD	LLOQ
y = 2.2502x + 0.0499	0.9995	0.01~2.0	0.0028	0.0091

LOD, limit of detection; LLOQ, lower limit of quantification; R^2^, coefficients of determination; SFN, sulforaphane.

**Table 2:** Intra- and inter-day precision and accuracy for quantification for SFN in rat plasma by the HPLC method.

Spike Amount (µg/mL)	Intra-Day (RSD, %) ^1^	Inter-Day (RSD, %)	Intra-Day (Accuracy, %) ^2^	Inter-Day (Accuracy, %)
0.01	7.95	8.14	93.41 ± 6.41	94.21 ± 7.14
0.03	7.27	7.69	92.15 ± 6.71	91.97 ± 8.19
0.8	3.04	3.41	100.17 ± 3.27	100.83 ± 3.57
2.0	1.57	2.22	98.94 ± 2.11	99.09 ± 2.58

^1^ RSD (%) = standard deviation of the concentration/mean concentration × 100. ^2^ Accuracy (%) = calculated concentration/theoretical concentration × 100.

**Table 3:** Recovery, matrix effect, and process efficiency of SFN and the IS from spiked rat plasma (*n* = 4).

Component	Spike Amount (µg/mL)	Recovery ^1^	Matrix Effect ^2^	Process Efficiency ^3^
SFN	0.03	85.31 ± 4.94	−2.87 ± 0.14	82.35 ± 5.89
	0.8	86.71 ± 1.24	−2.99 ± 0.16	84.10 ± 2.71
	2.0	87.41 ± 0.47	−2.37 ± 0.13	85.91 ± 0.83
IS	0.5	80.27 ± 2.18	−2.58 ± 0.21	78.14 ± 2.61

^1^ Recovery = (Response before extraction spiked sample/Response post-extracted spiked sample) × 100. ^2^ Matrix effect = (Response post-extracted spiked sample/Response non-extracted neat sample − 1) × 100. ^3^ Process efficiency = (Response before extraction spiked sample/Response non-extracted neat sample) × 100. IS, internal standard; SFN, sulforaphane.

**Table 4:** Stability test for SFN in rat plasma.

Condition Tested	0.03 µg/mL			0.8 µg/mL			2 µg/mL		
	Mean	RSD ^1^ (%)	RE ^2^ (%)	Mean	RSD (%)	RE (%)	Mean	RSD (%)	RE (%)
Short-term stability									
Freeze-thaw (−80 °C, 3 cycle)	0.028	3.74	−7.67	0.797	2.91	−0.38	2.013	1.84	0.65
Refrigerator (4 °C, 1 day)	0.025	3.12	−17.67	0.589	3.17	−26.38	1.731	1.56	−13.45
Freezer (−20 °C, 1 day)	0.027	3.39	−10.00	0.703	2.67	−12.13	1.959	1.58	−2.05
Freezer (−80 °C, 1 day)	0.033	2.95	9.67	0.751	3.10	−6.13	2.003	0.87	0.15
post-preparative stability (4 °C, 1 day)	0.030	2.34	−1.00	0.792	2.36	−1.00	2.084	1.41	4.20
post-preparative stability (4 °C, 1 week)	0.031	3.49	4.33	0.862	2.81	7.75	2.002	2.10	0.10
Long-term stability									
Freezer (−80 °C, 30 days)	0.032	3.81	7.00	0.780	2.90	−2.50	1.777	1.59	−11.15
Freezer (−20 °C, 30 days)	0.024	2.67	−19.67	0.768	3.13	−4.00	1.726	2.88	−13.70

^1^ RSD (%) = standard deviation of the concentration/mean concentration × 100. ^2^ RE (%) = calculated concentration/theoretical concentration × 100.

**Table 5:** Pharmacokinetic parameters of SFN after oral administration at doses of 10 or 20 mg/kg to rats (mean ± S.D., *n* = 4 rats).

Parameters	10 mg/kg	20 mg/kg
AUC_0–12h_ (μg·h/mL)	4.88 ± 0.89	9.85 ± 1.37
AUC_0–∞h_ (μg·h/mL)	6.25 ± 1.59	12.42 ± 2.36
AUMC (0–12 h)	21.85 ± 5.11	44.57 ± 7.42
MRT (0–12 h) (h)	4.45 ± 0.24	4.51 ± 0.13
t_1/2_ (h)	5.05 ± 0.91	5.47 ± 0.56
T_max_ (h)	1.00 ± 0.00	1.00 ± 0.00
CLz/F (L/h/kg)	1.67 ± 0.44	1.65 ± 0.32
Vz/F (L/kg)	11.83 ± 0.93	12.84 ± 1.14
C_max_ (μg/mL)	0.92 ± 0.09	1.67 ± 0.24

AUC, area under the curve; AUMC, area under the first moment curve; CLz/F, apparent oral clearance; Cmax, peak plasma concentration; MRT, mean residence time; t_1/2_, terminal; Tmax, time to reach Cmax; Vz/F, apparent volume of distribution.

## Data Availability

Not applicable.

## Sample Availability

Samples of the compounds are not available from the authors.
